# Increased expression of the chemokines CXCL1 and MIP-1α by resident brain cells precedes neutrophil infiltration in the brain following prolonged soman-induced status epilepticus in rats

**DOI:** 10.1186/1742-2094-8-41

**Published:** 2011-05-02

**Authors:** Erik A Johnson, Thuy L Dao, Michelle A Guignet, Claire E Geddes, Andrew I Koemeter-Cox, Robert K Kan

**Affiliations:** 1 Research Division, Pharmacology Branch, US Army Medical Research Institute of Chemical Defense (USAMRICD), Aberdeen Proving Ground, MD 21010, USA

## Abstract

**Background:**

Exposure to the nerve agent soman (GD) causes neuronal cell death and impaired behavioral function dependent on the induction of status epilepticus (SE). Little is known about the maturation of this pathological process, though neuroinflammation and infiltration of neutrophils are prominent features. The purpose of this study is to quantify the regional and temporal progression of early chemotactic signals, describe the cellular expression of these factors and the relationship between expression and neutrophil infiltration in damaged brain using a rat GD seizure model.

**Methods:**

Protein levels of 4 chemokines responsible for neutrophil infiltration and activation were quantified up to 72 hours in multiple brain regions (i.e. piriform cortex, hippocampus and thalamus) following SE onset using multiplex bead immunoassays. Chemokines with significantly increased protein levels were localized to resident brain cells (i.e. neurons, astrocytes, microglia and endothelial cells). Lastly, neutrophil infiltration into these brain regions was quantified and correlated to the expression of these chemokines.

**Results:**

We observed significant concentration increases for CXCL1 and MIP-1α after seizure onset. CXCL1 expression originated from neurons and endothelial cells while MIP-1α was expressed by neurons and microglia. Lastly, the expression of these chemokines directly preceded and positively correlated with significant neutrophil infiltration in the brain. These data suggest that following GD-induced SE, a strong chemotactic response originating from various brain cells, recruits circulating neutrophils to the injured brain.

**Conclusions:**

A strong induction of neutrophil attractant chemokines occurs following GD-induced SE resulting in neutrophil influx into injured brain tissues. This process may play a key role in the progressive secondary brain pathology observed in this model though further study is warranted.

## Background

Soman (pinacolyl methylphosphonofluoridate, GD) is a G-series nerve agent that rapidly and irreversibly binds to acetylcholinesterase, causing excess acetylcholine accumulation in the central and peripheral nervous systems, which results in cholinergic crisis. A consequence of this cholinergic crisis is rapid induction of status epilepticus (SE) that can continue unabated for many hours [1]. The duration of this seizure activity increases the magnitude of neuropathology [2,3] with the hippocampus, amygdala, thalamus and piriform cortex being the most severely affected [4,5]. Although initial injury occurs rapidly, a robust neuroinflammatory response can exacerbate damage to the brain over time. Neuroinflammation is a key factor in pathology development in other models of SE [6,7] as well as following nerve agent-induced seizure [8-11].

An early component of neuroinflammation is the recruitment and activation of circulating neutrophils to areas of injury. Neutrophil infiltration is an important step in the development of neuropathology following seizure [6,12,13]. Once through the blood-brain barrier, the respiratory burst of neutrophils can exacerbate the initial injury through indiscriminate protease damage to surrounding healthy tissues [14]. Infiltrating neutrophils are directed to and activated in injured brain regions by chemokines. For example, the chemokine (C-X-C motif) ligand 1 (CXCL1 or GRO KC) directs neutrophils to injured tissues [15] and propagates the neuroinflammatory response by inducing the synthesis of acute phase response cytokines interleukin (IL)-1, IL-6 and tumor necrosis factor-α in those cells [16]. Similarly, macrophage inflammatory protein (MIP)-1α functions to recruit and activate granulocytes (including neutrophils) in damaged brain regions [17-19]. Because inflammatory chemokines are up-regulated in many SE models [20-22], these factors likely play a role in this model as well.

Most studies of neuroinflammation following seizurogenic nerve agent exposure have centered on transcript changes [8,23,24] or limited protein changes [10]. Recently, however, we have reported the upregulation of multiple acute phase cytokines in this GD model [11]. In this study, we quantified the protein levels of the neutrophil chemoattractant and activating factors CXCL1, MIP-1α, granulocyte colony stimulating factor (G-CSF) and granulocyte-macrophage colony stimulating factor (GM-CSF), using multiplex immunoassays in brain tissue lysates following GD exposure up to 72 hours after SE onset. Additionally, cell-specific chemokine expression and neutrophil infiltration were investigated in damaged brain regions (i.e. piriform cortex, hippocampus and thalamus). CXCL1 and MIP-1α concentrations were significantly increased in all three brain regions investigated, while no change was observed in G-CSF or GM-CSF. CXCL1 and MIP-1α predominantly localized to neurons and either endothelial cells (CXCL1) or microglia (MIP-1α). Expression also preceded and positively correlated to significant neutrophil infiltration in these brain regions. These data are the first to show upregulation and cellular expression of chemokines and the ensuing influx of neutrophils in damaged brain regions following GD-induced SE.

## Methods

### GD seizure model

This model has been described previously [11,25]. Briefly, adult male Sprague-Dawley rats (Charles River Laboratories, Wilmington, MA; CRL: CD[SD]-BR, 250 - 350 g) were treated with HI-6 dichloride (1-(((4-(aminocarbonyl)pyridinio) methoxy)methyl)-2-((hydroxyimino)methyl)pyridinium dichloride)(BN44621, Starks Associates, Buffalo, NY; 125 mg/kg, i.p.) 30 minutes prior to GD administration and with atropine methyl nitrate (AMN, Sigma-Aldrich, St. Louis, MO; 2.0 mg/kg, i.m.) 1 minute after GD administration. Vehicle control animals received HI-6, AMN and saline, while naïve animals received no injections. GD (GD-U-2323-CTF-N, purity 98.8 wt%) was diluted in saline at the United States Army Medical Research Institute of Chemical Defense (USAMRICD) and administered subcutaneously (1.6 LD_50 _= 180 μg/kg). The experimental protocol was approved by the Animal Care and Use Committee at USAMRICD, and all procedures were conducted in accordance with the principles stated in the Guide for the Care and Use of Laboratory Animals (National Research Council, 1996), and the Animal Welfare Act of 1966 (P.L. 89-544), as amended. The animal care program at this institute is fully accredited by the Association for Assessment and Accreditation of Laboratory Animal Care International.

### Multiplex bead array immunoassay

As previously described [11], piriform cortex, hippocampus and thalamus brain tissue samples were procured from experimental and vehicle control animals at 0.5, 1, 3, 6, 12, 24, 48 or 72 hours after onset of convulsions. Tissue lysates were produced by first rinsing the excised tissue with cold PBS followed by snap freezing in liquid nitrogen. A ratio of 1 ml ice-cold triple detergent lysis buffer containing a Complete™ protease inhibitor cocktail (Roche Biochemicals, Indianapolis, IN) to 50 mg of frozen tissue was used for homogenization. Two 30 sec pulses on a mini Beadbeater (Biospec Products Inc., Bartlesville, OK) using 3.2 mm stainless steel beads were used to homogenize the tissue. Samples were centrifuged at 8000 × g for 5 minutes to separate the lysate from the tissue pellet. Rat cytokine multiplex bead immunoassay kits were used to quantify the concentrations of CXCL1 (GRO KC), MIP-1α, G-CSF and GM-CSF (LINCO Research, St. Charles, MO). Individual standard curves were generated in duplicate using the supplied reference chemokine concentrations according to the manufacturer's instructions. A volume of 25 μl of sample (94 ± 8 μg protein) per well, assayed in duplicate, was used for data generation. The plate was read on a Bioplex™ 100 instrument (Bio-Rad Laboratories, Hercules, CA) and analyzed with either BioRad or STaRStation software (Applied Cytometry, Sacramento, CA). Values that were calculated by the assay to be below the minimum detectable concentration (MinDC) for that particular analyte were conservatively estimated to be the MinDC value minus 0.01 pg/ml for statistical analysis. The number of replicates for the experimental samples are as follows: piriform cortex, n = 6 for each time point and naïve; hippocampus, n = 6 for each time point except for naïve (n = 5), 6 hr (n = 5) and 24 hr (n = 7); and thalamus, n = 5 for each time point and naïve except for 0.5 hr (n = 6), 6 hr (n = 4), 12 hr (n = 3), 24 hr (n = 6) and 48 hr (n = 6). Time matched vehicle controls (n = 3 per time point) were analyzed individually and condensed into a single vehicle control comparison group when no significant statistical difference was found between these samples over time by analyte or brain region.

### Immunohistochemistry (IHC)

Separate from the animals used in the multiplex bead array immunoassay, experimental, vehicle control and naïve animals were deeply anesthetized and perfused with isotonic saline followed by 4% paraformaldehyde via cardiac puncture. Brains were processed and sectioned at 40 microns as previously described [11]. The 12 hour time point was selected based on the peak expression times of the analytes from the multiplex assays. Free float fluorescent IHC labeling was conducted as previously described [26]. The antibodies used were as follows: rabbit anti-Gro α (CXCL1) (1:100; ab9772), rabbit anti-MIP-1α (1:500, ab9781) and mouse anti-rat endothelial cell antigen (RECA,1:1000; ab9774) from Abcam (Cambridge, MA), mouse anti-NeuN to label neurons (1:1000; MAB377) and mouse anti-CD11b to label microglia and macrophages (1:1000; CBL1512) from Chemicon (Temecula, CA), and mouse anti-GFAP to label astrocytes (1:1000; MS-280-P) from NeoMarkers (Fremont, CA). Alexafluor™ fluorescent-tagged secondary and tertiary antibodies (Molecular Probes, Eugene, OR) were used for visualization. Tissue sections labeled with only secondary and tertiary antibodies were used as controls. Sections were viewed and digitally captured with an Olympus BX51 microscope equipped with an Olympus DP-70 high-resolution color CCD digital camera (Opelco, Dulles, VA). An Olympus BX61 equipped with a DSU spinning disk confocal system and DP-70 CCD camera and a Zeiss LSM 700 confocal microscope were used for subsequent IHC micrographs to confirm same cell co-localization. Images of 40-μm tissues were acquired using a z step interval of 1 μm and analyzed using Slidebook™ (Olympus) or Zen 2009 (Zeiss) software. Publication images were compiled using Adobe Photoshop CS digital image software. Color levels and background labeling were reduced and evened using the "levels" tool. All input levels (0-255) were normalized in the RGB channel as follows: highlight input levels were set at the peak of the image histogram, midtone levels were set at 0.8 and shadow levels were set either at the edge of the histogram closest to 255 or at 180, whichever was greater. This technique was successful at reducing background while not oversaturating specific labeling. For all time points, n = 3.

### Quantitative Stereology

Sections were labeled with Mayer's hematoxylin/eosin-phloxine stain. Infiltrating neutrophils were visually identified by the user and quantified within the piriform cortex, hippocampus and thalamus using Stereologer 2000 software (Stereology Resource Center, Chester, MD) on an Olympus BX51 microscope equipped with an IK-C44H CCD Toshiba camera (Imaging Planet, Goleta, CA). Four to eight sections from tissue slabs of approximately 1440 to 3300 μm in length were used for counting in each case. Estimates used the optical fractionator method. For each tissue section analyzed, section thickness was assessed empirically and guard zones 2 μm thick were used at the top and bottom of each section. The tissue regions were outlined using 10 × magnification, and cells were counted using 40 × magnification. Approximately 50% of the outlined region was analyzed using a systematic random sampling design with a counting frame size of 175 μm and a disector height of 7 μm. The coefficients of error (CE) were calculated by the software and maximum CE was set at 0.1600.

### Statistical Analysis

Immunoassay data were evaluated by one-way ANOVA with a post-hoc Dunnett's analysis and expressed in pg/ml. Neutrophil stereology data were evaluated by one-way ANOVA with a post-hoc Newman-Kuel analysis and expressed as cells/mm^3^. A Pearson's correlation coefficient using a one measurement time lag between CXCL1 or MIP-1α concentration and neutrophil infiltration was also calculated. The one measurement time lag was used due to the many downstream molecular events that occur following neutrophil exposure to these chemokines that allow the neutrophil to traverse the vasculature into the injured tissue. Values are expressed as mean ± SEM. Differences were considered significant at the level of p ≤ 0.05.

## Results

### Brain concentrations of CXCL1 significantly increase in response to GD-induced SE

Temporal and regional changes in CXCL1, MIP-1α, G-CSF and GM-CSF protein concentrations were determined using a bead-based multiplex immunoassay on tissue lysates from the piriform cortex, hippocampus and thalamus. CXCL1 and MIP-1α significantly increased in all brain regions investigated. G-CSF and GM-CSF concentrations did not significantly change and were not analyzed further (data not shown).

**Figure 1:**
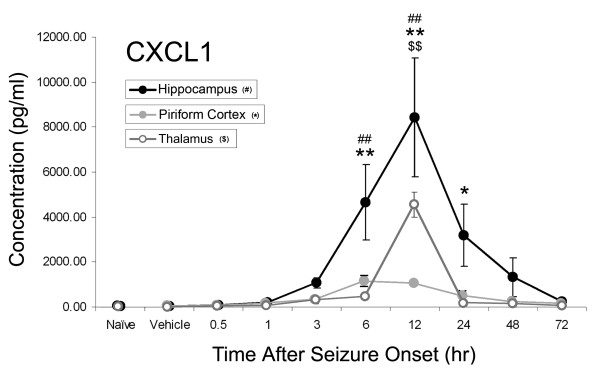
**CXCL1 increases in rat brain after GD-induced SE**. Concentrations of CXCL1 peak at 6 hours in the piriform cortex (solid gray line) and 12 hours in the hippocampus (solid black line) and thalamus (open gray line). Data are given as pg/ml of tissue lysate and reported as mean ± SEM. Data were analyzed using a one-way ANOVA with a post-hoc Dunnett's analysis comparing to vehicle control. (^## ^p < 0.01 hippocampus, * p < 0.05, ** p < 0.01 piriform cortex, ^$$ ^p < 0.01 thalamus).

CXCL1 concentrations significantly increased in all three brain regions (Figure 1). The highest concentrations were in the hippocampus, where concentrations significantly increased by 6 hours (4674 ± 1504 pg/ml) and peaked by 12 hours (8441 ± 2152 pg/ml vs. 58 ± 6 pg/ml in vehicle controls) following GD-induced SE. In the piriform cortex, CXCL1 levels peaked at 6 hours (1164 ± 195 pg/ml) and remained significantly elevated up to 24 hours (501 ± 176 pg/ml) compared to vehicle controls (49 ± 9 pg/ml). In the thalamus, CXCL1 concentration became significant at 12 hours compared to controls (4571 ± 643 pg/ml vs. 50 ± 3 pg/ml).

### CXCL1 is expressed by neurons and endothelial cells

**Figure 2:**
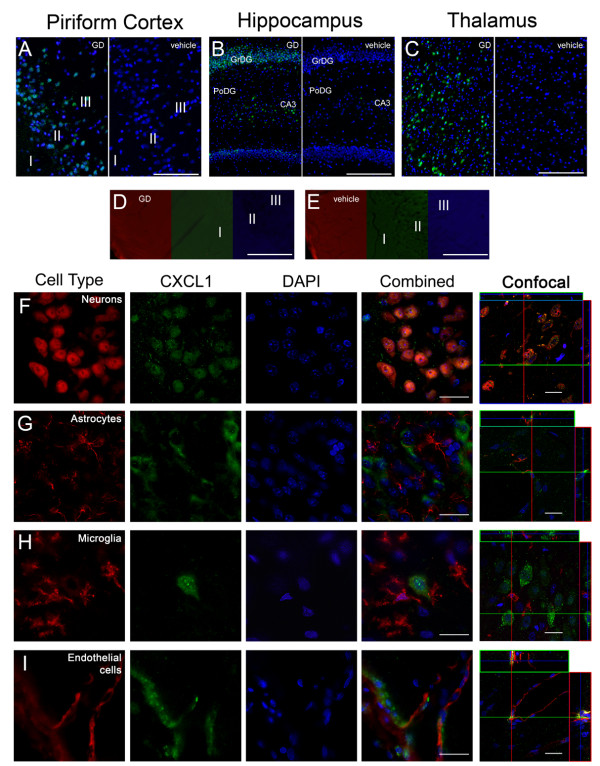
**CXCL1 is expressed in neurons and, to a much lesser extent, in endothelial cells after GD-induced SE**. Prominent CXCL1 (A-I, green) immunolabeling is present in the piriform cortex, hippocampus and thalamus 12 hours after GD-induced SE (A, B & C; left). CXCL1 is absent in vehicle controls of these same regions (A, B & C; right). In the piriform cortex (A), labeling is observed primarily in layers II and III. In the hippocampus (B), labeling was primarily confined to the CA3 pyramidal layer and granular layer of the dentate gyrus (GrDG) but not evident in the polymorphic layer of the dentate gyrus (PoDG). CXCL1 was located to the laterodorsal and lateral posterior nuclei of the thalamus (C). Labeling was absent in the secondary controls for both 12-hour GD-exposed (D) and vehicle control tissues (E), exemplified by the piriform cortex. Neurons (F, red) and CXCL1 were often found to co-localize (F, yellow). Co-localization was not observed in hypertrophic astrocytes (G, red) or activated microglia (H, red) and was limited in endothelial cells (I, yellow). DAPI (A-I, blue) was used to label the nuclei of each cell. Scale bar: 250 μm (A-E), 50 μm and 20 μm (F-I) for regular and confocal fluorescent microscopy respectively; n = 7 for 12-hour, n = 4 for vehicle controls.

Twelve hours following GD-induced SE, CXCL1 immunolabeling was present in the piriform cortex (Figure 2A, left), hippocampus (dentate gyrus shown; Figure 2B, left) and thalamus (Figure 2C, left), while CXCL1 labeling was absent in vehicle controls in the same regions (Figure 2A, B, &2C, right). Specific labeling was also absent in secondary only controls at the 12-hour time point (Figure 2D) and in vehicle controls (Figure 2E) as exemplified by the piriform cortex. In the piriform cortex, CXCL1-positive cells were found predominantly in layer II but also in layer III. In the hippocampus, CXCL1-positive cells were found primarily in the granular layer of the dentate gyrus (GrDG) and the CA3 pyramidal layer closest to the dentate gyrus. CXCL1-positive cells were also found in the laterodorsal and lateral posterior nuclei of the thalamus. To identify these cells, sections were co-labeled with antibodies specific for neurons, astrocytes, microglia and endothelial cells and for CXCL1. CXCL1 immunoreactivity was found in neuronal populations in the regions mentioned above (Figure 2F). CXCL1 diffusely labeled the cytoplasm in these cells with interspersed fine punctate labeling. No co-localization was observed between CXCL1 and astrocytes (Figure 2G) or microglia (Figure 2H) regardless of state of activation. Co-localization was limited in endothelial cells, though a prevalent, but not exclusive, association between CXCL1 and the vasculature was often observed (Figure 2I).

### Brain concentrations of MIP-1α significantly increase in response to GD-induced SE

**Figure 3:**
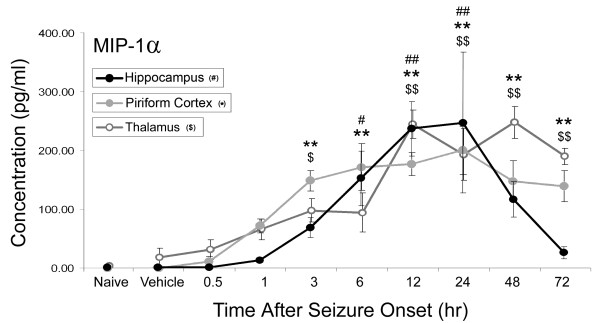
**MIP-1α Increases in Rat Brain after GD-induced SE**. MIP-1α concentrations significantly increase in the hippocampus, piriform cortex and thalamus following GD-induced SE. MIP-1α concentrations peak at 24 hours after seizure onset in both the hippocampus (solid black line) and piriform cortex (solid gray line). In the thalamus (open gray line), there is a double peak at 12 and 48 hours after seizure onset. Data are given as pg/ml of tissue lysate reported as mean ± SEM. Data were analyzed using a one-way ANOVA with a post-hoc Dunnett's analysis comparing to vehicle control. (^# ^p < 0.05, ^## ^p < 0.01 hippocampus; ** p < 0.01 piriform cortex; ^$ ^p < 0.05, ^$$ ^p < 0.01 thalamus).

Significant concentration increases were observed for MIP-1α protein in all three brain regions following GD-induced seizure (Figure 3). MIP-1α concentrations significantly increased in the hippocampus at 6 hours (152 ± 42 pg/ml), peaked at 24 hours (247 ± 90 pg/ml) and then rapidly decreased by 48 hours after SE onset compared to vehicle controls (2.06 ± 0.05 pg/ml). In the piriform cortex, MIP-1α concentrations significantly increased at 3 hours (149 ± 14 pg/ml), peaked at 24 hours (200 ± 34 pg/ml) and remained elevated through the 72-hour endpoint (139 ± 22 pg/ml) compared to vehicle controls (<1.94 pg/ml, MinDC). The pattern in the thalamus was different still, where a double peak was observed at 12 hours (245 ± 28 pg/ml) and 48 hours (248 ± 22 pg/ml) compared to controls (18 ± 11 pg/ml).

### MIP-1α is expressed by neurons and microglia

**Figure 4:**
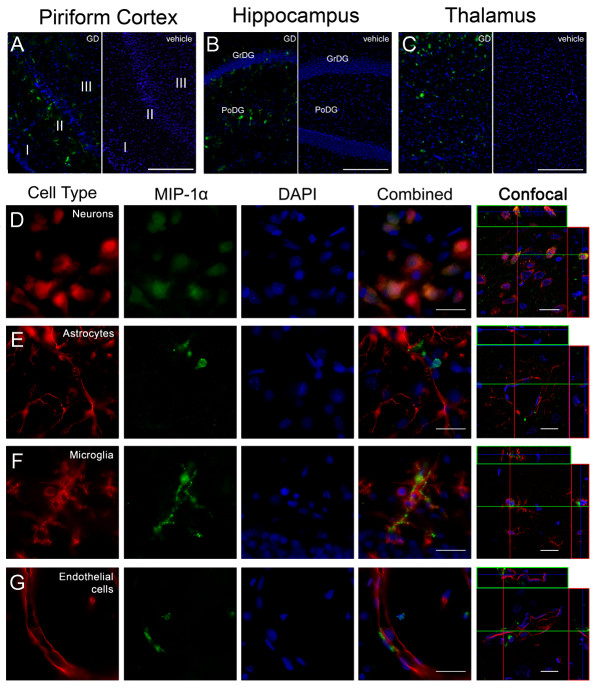
**MIP-1α is primarily expressed by neurons and dystrophic microglia after GD-induced SE**. MIP-1α immunolabeling (A-G, green) is present in the piriform cortex, hippocampus and thalamus 12 hours after GD-induced SE (A, B & C; left) but absent in vehicle controls of these same regions (A, B & C; right). In the piriform cortex (A), labeling is observed primarily in layers II and III. In the hippocampus (B), labeling was less robust and found primarily in the CA1 and CA3 pyramidal layers as well as in the polymorphic layer of the dentate gyrus (PoDG) but not the granular layer of the dentate gyrus (GrDG). MIP-1α labeling was also less robust in the thalamus (C) and was found primarily in the laterodorsal and lateral posterior nuclei. Neurons (D, red) and MIP-1α frequently co-localized (D, yellow), while no co-localization with astrocytes (E, red) was observed. MIP-1α was primarily expressed by microglia with a dystrophic morphology (F, red). Limited co-localization was observed in endothelial cells (G, red). Scale bar: 250 μm (A-C), 50 μm and 10 μm (D-F) for regular and confocal fluorescent microscopy respectively; n = 9 for 12-hour and n = 4 for vehicle controls.

Twelve hours following seizure onset, MIP-1α immunolabeling was present in piriform cortex (Figure 4A, left), hippocampus (dentate gyrus shown; Figure 4B, left) and thalamus (Figure 4C, left) but not in vehicle controls (Figure 4A, B &4C, right). Weak to moderate diffuse co-localization with neurons (Figure 4D) was observed in layers II and III of the piriform cortex, the CA1 and CA3 pyramidal layers of the hippocampus and the polymorphic (PoDG) but not the granular layer (GrDG) of the dentate gyrus. Astrocytes were not found to express MIP-1α in any region observed (Figure 4E). Activated microglia strongly expressed MIP-1α in all regions investigated. These cells had a myriad of morphological features including hypertrophy, spheroid shape, blebbing and dystrophy. Specifically, MIP-1α shows a high degree of cellular localization with the dystrophic microglial morphology (Figure 4F). Lastly, MIP-1α-positive cells were closely associated with large blood vessel endothelial cells in the piriform cortex and thalamus but co-localization was not observed (Figure 4G).

### Neutrophil influx positively correlates with chemokine expression

**Figure 5:**
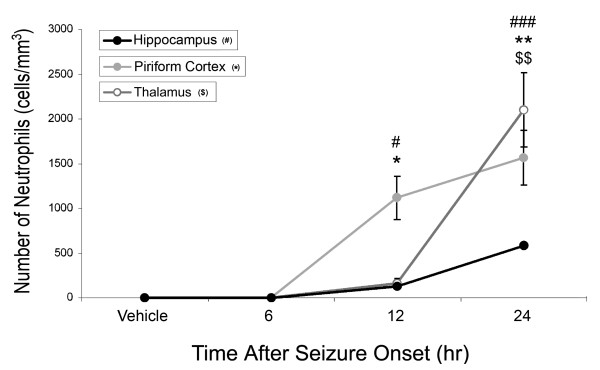
**Neutrophil infiltration occurs following significant CXCL1 expression in injured brain regions**. Significant increases in neutrophils were observed in the hippocampus (solid black line) and piriform cortex (solid gray line) at 12 and 24 hours following SE onset. Significant increases were observed in the thalamus (open gray line) at 24 hours only. Data are given as cells/mm^3 ^of tissue reported as mean ± SEM. Data were analyzed using a one-way ANOVA with a post-hoc Newman-Kuel analysis (^# ^p < 0.05, ^### ^p < 0.001 in hippocampus; ** p < 0.01 in piriform cortex; ^$$ ^p < 0.01 in thalamus; n = 3 for all brain regions and time points).

To determine whether neutrophil recruitment correlates to increases in CXCL1or MIP-1α, neutrophil counts in the piriform cortex, hippocampus and thalamus were quantified using stereological techniques and correlated to CXCL1 and MIP-1α concentration data using a one measurement time lag with Pearson's correlation analysis. Neutrophil infiltration significantly increases in all three observed brain regions following GD-induced SE (Figure 5). No neutrophils were found in vehicle controls in any brain region (0 ± 0 cells/mm^3^). In the piriform cortex, neutrophil infiltration significantly increased at 12 (1,117 ± 485 cells/mm^3^) and 24 hours (1,565 ± 618 cells/mm^3^) but not 6 hours (3 ± 5.8 cells/mm^3^) compared to vehicle. Significant, though less robust, neutrophil infiltration was also observed in the hippocampus at 12 (128 ± 85 cells/mm^3^) and 24 hours (589 ± 10 cells/mm^3^) but not 6 hours (0 ± 0 cells/mm^3^) compared to vehicle. In contrast, neutrophils in the thalamus significantly increased only at 24 hours (2,098 ± 824 cells/mm^3^) and not at 12 hours (158 ± 90 cells/mm^3^). Pearson's correlation analysis revealed a positive correlation between CXCL1 concentration and neutrophil infiltration (offset by one time point) in the piriform cortex, hippocampus and thalamus. For MIP-1α, a less robust positive correlation existed in the hippocampus and thalamus compared to CXCL1. No significant correlation was observed in the piriform cortex (Table 1).

**Table 1:** CXCL1 and MIP-1α expression positively correlates with the infiltration of neutrophils into injured brain.

	Brain Region	Pearson's r	P value	95% CI
**CXCL1**	Piriform cortex	0.945	0.015	0.377 to 0.996
	
	Hippocampus	0.946	0.015	0.383 to 0.996
	
	Thalamus	0.999	<0.001	0.978 to 0.999

**MIP-1α**	Piriform cortex	0.728	>0.05	-0.431 to 0.980
	
	Hippocampus	0.943	0.016	0.360 to 0.996
	
	Thalamus	0.954	0.012	0.452 to 0.997

## Discussion

Neuroinflammation is almost ubiquitous following brain injury, though little is known about this process following damage caused by GD-induced SE. As part of the inflammatory process, resident and systemic inflammatory cells migrate to areas of injury guided by concentration gradients of chemokines and growth factors. This study describes the temporal and regional protein changes of four neutrophil activating and chemotactic factors in the brain, the expression of significantly upregulated factors in resident brain cells, quantification of neutrophil infiltration into the brain, and the correlation between chemokine expression and neutrophil infiltration. Significant expression of two chemokines, CXCL1 and MIP-1α, immediately preceded neutrophil infiltration in brain regions damaged by SE (i.e., the piriform cortex, hippocampus and thalamus). Both chemokines were primarily expressed by neurons; however, CXCL1 was also expressed in endothelial cells, and MIP-1α was also expressed in activated microglia. These data are the first to show the temporal, regional and cellular protein expression of chemokines, consequent neutrophil infiltration and the relationship between these two events following nerve agent exposure and subsequent SE.

Of all the resident brain cell types, neurons appear most susceptible to GD-induced SE damage as shown by substantial neuronal cell death in the piriform cortex, thalamus and portions of the hippocampus [5,27]. Therefore, it is not surprising that neurons become the focal point of the inflammatory response. In fact, the neurons most vulnerable to GD-induced SE, including those in layer II of the piriform cortex [28], strongly expressed both CXCL1 and MIP-1α. Injured neurons have the ability to produce chemokines to recruit and activate inflammatory cells following injury [29-31], and we have now shown expression of CXCL1 and MIP-1α in the GD-induced SE model as well.

Astrocytes did not express CXCL1 or MIP-1α in any brain region despite concurrent neuronal injury. CXCL1 expression by astrocytes does occur following various central nervous system (CNS) insults [32-34], and this expression appears to be dependent on neuronal damage [35]. Similarly, MIP-1α is expressed by astrocytes in SE [36] and experimental autoimmune encephalomyelitis models [37,38]. Though it is unknown exactly why chemokine expression in this model is incongruent with other CNS injury paradigms, it is apparent that chemokine expression is likely insult specific [39], and neutrophil recruitment may not be the main function of astrocytes in this model or at this point in pathology progression. Further, cytokine expression is prominent in both astrocytes (IL-6) and microglia (IL-1) in this model and may function to modulate the neuroinflammatory process rather than to recruit inflammatory cells [11].

Despite a lack of expression in most microglia, MIP-1α was expressed by a number of activated microglia and prominently expressed by those with a dystrophic morphology. MIP-1α expression by active microglia following brain injury has been previously observed [40], though little is known about dystrophic microglia or the expression of inflammatory factors by this morphological type. It is known that dystrophic microglia appear exclusively in progressive neurodegenerative disease states such as Alzheimer's and Huntington's disease and are indicative of concurrent and subsequent neuronal degeneration [41-43], a condition that is accelerated in this model. We have previously shown that another important neutrophil chemoattractant [44] and upregulator of neutrophil infiltration endothelium adhesion molecules [45], IL-1β, was also localized to dystrophic microglia [11]. Therefore, dystrophic microglia appear to have a prominent role in the recruitment and activation of neutrophils following prolonged SE induced by GD.

Lastly, significant CXCL1 expression precedes a significant influx of neutrophils into vulnerable brain regions (<6 hours in the piriform cortex and hippocampus and <12 hours in the thalamus; Figures 1 &5). A less definitive positive correlation exists for MIP-1α, likely because MIP-1α is highly pleiotropic and also modulates the chemotaxic and activation properties of other leukocyte cell types [46,47]. Though there is a strong positive correlation between CXCL1 concentration and consequent neutrophil influx, this relationship does not appear to be proportional. For example, while we observed the highest concentrations of CXCL1 in the hippocampus, this region had the fewest number of infiltrating neutrophils. In contrast, the piriform cortex had the lowest concentration of CXCL1 but had some of the highest numbers of infiltrating neutrophils.

Though little is known about regional differences in brain chemokine expression, there are several variables that may influence the relationship between chemokine concentration and neutrophil infiltration. First, region specific neutrophil infiltration may rely on other cytokine induced neutrophil chemoattractant (CINC) family members. For example, CXCL1, also known as CINC-2β, was not found to be a major contributing factor in brain neutrophil infiltration following direct IL-1β injection into the brain compared to CINC-1 and CINC-2α [48]. It should be noted, however, that individual chemokine involvement is likely injury specific and these CINCs may not be active in this model. Second, differential IL-1β brain expression may play a role. We have previously documented regional differences in brain IL-1β concentration, an important promoter of neutrophil adhesion, in this model [11]. No significant expression of IL-1β was observed in the hippocampus whereas significant increases in IL-1β were observed in the piriform cortex and thalamus that correspond to neutrophil influx. However, IL-1α, an IL-1 isoform that can similarly increase CXCL1 expression and cellular adhesion molecules [49], was significantly increased and may serve a similar role as IL-1β in this model. Lastly, differential expression of CXCL1 receptors, CXCR1 and CXCR2, and the associated vascular cell surface glycosaminoglycans (GAGs), may account for the observed discrepancy between CXCL1 expression and neutrophil infiltration. GAGs are essential for forming chemotactic gradients [50] and affect chemokine binding to their associated G-protein-coupled receptors [51]. Because different chemokines bind with varying affinities to different GAGs [50] and GAG and CXCL1 receptor expression are highly dependent on the location, type and subset of the cell [52-56], varying rates of neutrophil infiltration are possibly at different neuroinflammatory foci dictated by these complex interactions.

## Conclusion

In conclusion, we have shown that concentrations of CXCL1 and MIP-1α significantly increase in the brain of rats following GD-induced SE. We have also identified specific cell types that express these factors; neurons and endothelial cells primarily express CXCL1, while neurons and dystrophic microglia primarily express MIP-1α. Neutrophil infiltration significantly increases in regions where CXCL1 and MIP-1α expression and neuronal death occur. Lastly, expression of these chemokines precedes neutrophil infiltration, consistent with their chemotactic properties, but infiltration is not necessarily proportional to chemokine concentration. These data suggest a strong activation and recruitment of neutrophils to areas of brain damage modulated, at least partially, by CXCL1 and MIP-1α expression by injured neurons, microglia and endothelial cells following GD-induced SE.

## List of abbreviations

CNS: central nervous system; GD: soman; SE: status epilepticus; MIP-1α: macrophage inflammatory protein 1α; CXCL1: chemokine (C-X-C motif) ligand 1; IL: interleukin; G-CSF: granulocyte colony stimulating factor; GM-CSF: granulocyte-macrophage colony stimulating factor; IHC: immunohistochemistry; MinDC: minimum detectable concentration.

## Competing interests

The authors declare that they have no competing interests.

## Authors' contributions

EAJ and RKK both participated in developing the study concept and experimental design. EAJ analyzed data, wrote the manuscript and participated in acquisition of data. TLD, MAG, CEG and AIKC acquired and analyzed data and contributed to the writing of the manuscript. All authors have read, edited and approved the final manuscript.
